# Augmentation of the anticancer activity of CYT997 in human prostate cancer by inhibiting Src activity

**DOI:** 10.1186/s13045-017-0485-0

**Published:** 2017-06-12

**Authors:** Yong Teng, Yafei Cai, Wenhu Pi, Lixia Gao, Chloe Shay

**Affiliations:** 1 Department of Oral Biology, Augusta University, Augusta, GA 30912 USA; 2 Georgia Cancer Center, Augusta University, 1120 15th Street, Augusta, GA 30912 USA; 3 Department of Biochemistry and Molecular Biology, Augusta University, Augusta, GA 30912 USA; 4 College of Animal Science and Technology, Nanjing Agricultural University, Nanjing, 210095 China; 5 Department of Radiation Oncology, Indiana University, Indianapolis, IN 46202 USA; 6 Department of Pediatrics, Emory Children’s Center, Emory University, Atlanta, GA 30322 USA

**Keywords:** CYT997, Prostate cancer, Dasatinib, Src, Anticancer, Synergistic treatment

## Abstract

**Background:**

Abnormalities of tubulin polymerization and microtubule assembly are often seen in cancer, which make them very suitable targets for the development of therapeutic approach against rapidly dividing and aggressive cancer cells. CYT997 is a novel microtubule-disrupting agent with anticancer activity in multiple cancer types including prostate cancer. However, the molecular mechanisms of action of CYT997 in prostate cancer have not been well characterized.

**Methods:**

Src knockdown cells were achieved by lentiviral-mediated interference. The drug effects on cell proliferation were measured by MTS. The drug effects on cell viability and death were determined by Cell Titer-Glo® Luminescent cell viability kit and flow cytometry with Zombie Aqua™ staining. The drug effects on apoptosis were assessed by Cell Death Detection Elisa kit and Western blot with a cleaved PARP antibody. The drug effects on cell invasion were examined by Matrigel-coated Boyden chambers. Oxidative stress was detected by DCFH-DA staining and electrochemical biosensor. Mouse models generated by subcutaneous or intracardiac injection were used to investigate the in vivo drug efficacy in tumor growth and metastasis.

**Results:**

CYT997 effectively inhibited proliferation, survival, and invasion of prostate cancer cells via blocking multiple oncogenic signaling cascades but not the Src pathway. Inhibition of Src expression by small hairpin RNA or inactivation of Src by dasatinib increased the CYT997-induced cytotoxicity of in vitro. Moreover, the combination of dasatinib and CYT997 exhibited a superior inhibitory effect on tumor growth and metastasis compared with either of the drugs alone.

**Conclusion:**

Our findings demonstrate that blockage of Src augments the anticancer effect of CYT997 on prostate cancer and suggest that co-treatment of dasatinib and CYT997 may represent an effective therapeutic regimen for limiting prostate cancer.

**Electronic supplementary material:**

The online version of this article (doi:10.1186/s13045-017-0485-0) contains supplementary material, which is available to authorized users.

## Background

Prostate cancer, known as carcinoma of the prostate, is the most commonly diagnosed solid tumor among men. Despite new technology has enabled medical researchers to develop more reliable, less invasive screening and treatment methods, prostate cancer still remains the second leading cause of cancer-related death in male [1]. The failure of clinical treatment in patients with prostate cancer is often due to the heterogeneous nature of the disease. Comparative understanding of genetic pathways involved in the development and progression of prostate cancer may help define novel therapeutic targets or/and identify treatment regimens that are very likely to provide therapeutic benefit to patients.

Disrupting normal function of microtubules can engage the spindle checkpoint and arrest cell cycle progression at mitosis, leading to cell death [2]. Therefore, targeting microtubules is one of the efficient strategies for cancer treatment. A bunch of clinical chemo drugs, such as vinblastine, docetaxel, and paclitaxel, have potential to suppress microtubule dynamics without affecting microtubule polymer mass [2–4]. Although these microtubule-targeting agents (MTAs) are widely used to treat different types of human cancers [5–7], substantial drawbacks such as narrow therapeutic windows and lack of oral bioavailability still remain [8]. Moreover, the potential side effects (e.g., neural toxicity) and cardio-vascular events (e.g., thromboembolism) largely limit therapeutic efficacy of MTA as single agents against cancer. To overcome these clinical problems, many research efforts have concentrated on developing novel MTAs. CYT997 is a new microtubule-disrupting agent screened from Cytopia’s small molecule library, which has been proven to possess varying degrees of activity against cancers through inhibiting tubulin polymerization and disrupting cellular microtubules [9–11]. In phase I clinical trials, the safety, efficacy, and pharmacokinetics of CYT997 in cancer patients have been investigated [9].

Src, a non-receptor tyrosine kinase, has been implicated in a variety of different cancer types as well as in progression to malignancy [12–14]. Activation of Src is associated with its translocation to the plasma membrane in which Src interacts with a number of important effectors in response to extracellular signals. Regardless of which mechanism for Src activation, Src promotes numerous properties associated with metastatic potential once it is phosphorylated on tyrosine residues [12, 13]. It has been reported that Src trafficking requires both microtubules and actin polymerization [15], which may link Src functions to MTAs. In hormone-dependent cancers (e.g., breast and prostate cancer), steroid hormones trigger association of the androgen receptor (AR)-estradiol receptor (ER) complex with Src. A synthetic 10 amino-acid peptide that mimics the sequence of the SH3 domain-mediated binding of Src can prevent the AR/ER complex from associating with Src and inhibit the growth of LNCaP xenografts established in nude mice [16]. A study from Dr. Migliaccio’s group shows that targeting the AR domain involved in AR/Src association impairs EGF signaling in human fibrosarcoma HT1080 cells [17]. Preclinical studies also established a role of Src in progressive stages of prostate cancer and cross talk between Src and AR signaling [18]. AR can also activate MAP kinase (MAPK) through an early and a late response pathway in a cell type-dependent manner [19]. The combination of the Src inhibitor PP2 and antiandrogen Casodex does not further decrease the invasion of the LNCaP derivative C4-2 cells [20], suggesting that Src-assisted invasion may involve nongenomic AR actions whereby MAPK axis stimulation by AR signaling. Recent studies further reported that the Src inhibitors (e.g., dasatinib, saracatinib, and bosutinib) can inhibit motility, migration, and invasion of androgen-dependent and androgen-independent prostate cancer cells in a dose-dependent manner [21, 22].

In this study, we show that CYT997 effectively inhibits proliferation, viability, and invasion of prostate cancer cells. Genetic or pharmacological blockage of Src sensitizes prostate cancer cells towards CYT997 regardless of AR expression. Synergistic treatment with CYT997 and the Src inhibitor dasatinib exhibits a superior anticancer effect in mouse models of prostate cancer. These findings provide a molecular and cellular basis for CYT997 treatment, suggesting a clinical evaluation of CYT997 in combination with dasatinib for the treatment of patients with prostate cancer.

## Methods

### Cell lines, reagents, antibodies, and standard assays

Prostate cancer cell lines PC3, DU145, LNCaP, and 22Rv1 were obtained from ATCC and passage <5 were used in this study. LNCaP-derivative C4-2 and C4-2B cells were a gift from Dr. Daqing Wu (Georgia Cancer Center). All cells were maintained in Dulbecco’s modified Eagle’s medium (DMEM) containing 10% fetal bovine serum (FBS). Luciferase stable PC3 cells were generated by transduction of pGL4.5 vector (Promoga, Madison, WI) encoding the luciferase reporter gene luc2 and selection of hygromycin. pLKO.1 lentiviral vectors harboring small hairpin RNAs (shRNAs) targeting Src were obtained from Open Biosystems (Huntsville, AL). CYT997 and dasatinib were purchased from Selleckchem (Houston, TX). 2′,7′-Dichlorodihydrofluorescein diacetate (DCFH-DA) and D-Luciferin bioluminescent substrate were purchased from Sigma-Aldrich (St Louis, MO). Antibodies that recognize p62, p-AKT (Ser473), p-ERK1/2 (Thr202/Tyr204), p-STAT3 (Tyr705), p-Src (Tyr416), AKT, ERK1/2, STAT3, Src, LC3B I/II, and cleaved (c)-PARP were purchased from Cell Signaling Technology (Beverly, MA). β-Actin and Ki67 antibodies were purchased from Sigma-Aldrich (St Louis, MO) and Abcam (Cambridge, MA), respectively. Western blot, cell proliferation, and electrochemical detection were carried out as described previously [23–28]. Matrigel invasion assays were performed by transwells from BD biosciences (San Jose, CA) as described previously [23–26]. Briefly, 5 × 10^4^ serum-starved cells were seeded into Matrigel-coated Boyden chambers in the presence or absence of the indicated concentrations of CYT997, and DMSO containing 10% FBS was added to the lower chamber. After 24-h treatment, the membranes that contained invading cells were fixed in methanol and stained with 0.2% Crystal violet. The dye was dissolved in 10% acetic acid and read colorimetrically at 590 nm for quantification of invasion. Cell viability was determined by CellTiter-Glo® Luminescent cell viability assay (Promega, Madison, MI) and Zombie Aqua™ fixable viability kit (BioLegend, San Diego, CA). Flow cytometry data were analyzed using FlowJo software (Tree Star, Ashland, OR).

### Detection of apoptosis

The Cell Death Detection Elisa kit (Roche, Indianapolis, IN) was used to determine apoptosis by measuring mono- and oligonucleosomes in the lysates of apoptotic cells according to the manufacturer’s protocol. Briefly, the cells treated with different drugs were lysed and placed into a streptavidin-coated microplate and incubated with a mixture of anti-histone-biotin and anti-DNA-peroxidase. The amount of peroxidase retained in the immunocomplex was photometrically determined with 2,2′-azino-bis(3-ethylbenzothiazoline-6-sulphonic acid) (ABTS) as the substrate. Absorbance was measured at 405 nm (492 nm as reference wavelength).

### Animal models

Six-week-old male NSG (NOD.Cg*-Prkdc* ^*scid*^ *Il2rg* ^*tm1Wjl*^ */SzJ*) mice were purchased from the Jackson Laboratory (Bar Harbor, ME), and all animal experiments were approved by the Institutional Animal Care and Use Committee (IACUC) of Augusta University. To generate a xenotransplantation model, exponentially growing PC3 cells (1 × 10^6^ cells) were suspended in 100 μl PBS/matrigel (1:1) and injected subcutaneously into the right flanks of NSG mice. To generate an intracardiac model, NSG mice were injected via intracardiac with 1 × 10^6^ luciferase-containing PC3 cells.

### Drug administration and immunohistochemistry

Four days after PC3 cell implantation, mice were randomized to receive control vehicle and drug(s) (*n* = 5). The treatment groups received followed equal volume treatment of CYT997 (20 mg/kg), dasatinib (10 mg/kg), or in combination of CYT997 (20 mg/kg) and dasatinib (10 mg/kg), respectively. CYT997 was administered by garage twice per day for a total of 3 weeks, and dasatinib was intraperitoneally (i.p.) administered 5 days per week for a total of 4 weeks. The control mice were injected i.p. with 100 μl sterile saline. Tumor growth was measured externally every 4 to 7 days using vernier calipers as length × width^2^ × 0.52. The mice were sacrificed on treatment day 42, and the lungs were removed and processed for immunohistochemistry (IHC) with Ki67 antibody and pathological analysis by HE staining as described previously [29, 30]. For intracardiac models, mice were randomized for vehicle (sterile saline) or combined (20 mg/kg CYT997 with 10 mg/kg dasatinib) treatment. Mice were imaged for luciferase signal by an intraperitoneal injection of luciferin (15 μg in 100 μl PBS) every other week for 4 weeks using a Xenogen IVIS-200 In Vivo Imaging System (PerkinElmer, Waltham, MA).

### Statistical analysis

Treatment effects were evaluated using a two-tailed Student *t* test at each measurement time point. To assess the longitudinal effect of treatment, a mixed model was employed to test the overall difference across all groups as well as between each pair of groups during the whole study period. The data were presented as means ± SD from three or more independent experiments, and a *p* value less than 0.05 was considered significant.

## Results

### CYT997 inhibits proliferation, viability, and invasion of prostate cancer cells through blocking multiple signaling pathways

**Fig. 1:**
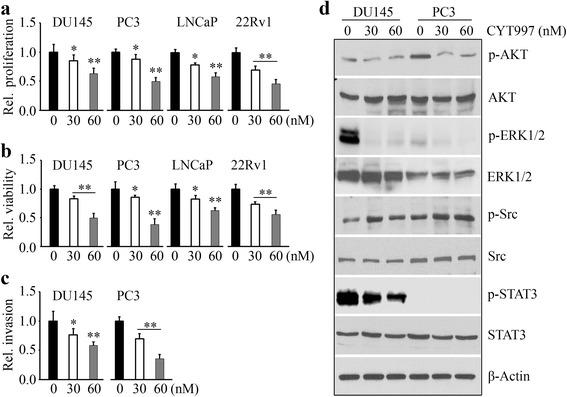
CYT997 exhibits potent cytotoxicity against prostate cancer cells in vitro. **a**, **b** 1 × 10^4^ prostate cancer cells (DU145, PC3, LNCaP, and 22Rv1) were seeded into 96-well plates and treated with the indicated concentrations of CYT997 for 48 h, and cell proliferation and viability were determined by MTS assays (**a**) and CellTiter-Glo® Luminescent cell viability assays (**b**), respectively. **c** DU145 and PC3 cells were seeded into Matrigel-coated Boyden chambers in the presence or absence of the indicated concentrations of CYT997 for 24 h, and invasion ability was quantitatively estimated by the absorbance value. **d** DU145 and PC3 cells were treated with CYT997 for 24 h, and cell lysates were collected for Western blot with the indicated antibodies

CYT997 has been tested against a panel of 16 cancer cell lines and displays broad cytotoxicity in vitro [10]. To evaluate the drug effects on prostate cancer, prostate cancer cell lines with different genetic background were treated with various concentrations of CYT997. AR are highly expressed in 22Rv1, LNCaP, and its derivatives (C4-2 and C4-2B), and they are androgen-responsive cell lines. In contrast, DU145 and PC3 have relatively low basal levels of AR which are unresponsive to androgen stimulation. CYT997 inhibited proliferation and viability of all these cells in a dose-dependent manner (Fig. 1a, b and Additional file 1: Figure S1), suggesting that CYT997 exhibits inhibitory effects on cancer growth and survival regardless of AR expression. DU145 and PC3 are highly invasive prostate cancer cells, and their invasion potential was determined to explore the effect of CYT997 on cell motility. Transwell invasion assays showed that CYT997 effectively decreased cell invasion (Fig. 1c), suggesting CYT997 may block metastasis of prostate cancer. To determine the possible mechanisms involved in mediating drug action, we examined multiple oncogenic signaling pathways by Western blot. This investigation revealed a decreased phospho-activation of AKT and ERK1/2 following CYT997 treatment (Fig. 1d), which demonstrates that CYT997 can simultaneously suppress PI3K/AKT and MAPK pathways. Phosphorylation of STAT3 in PC3 cells remained undetectable regardless of CYT997 treatment; however, a sharp decrease in activated STAT3 was observed in DU145 cells when exposed to CYT997 (Fig. 1d).

### CYT997 induces apoptosis in prostate cancer cells

**Fig. 2:**
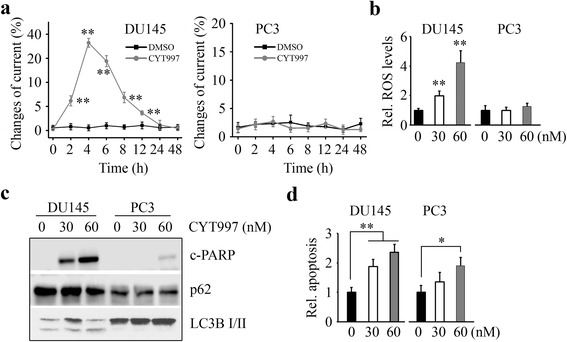
CYT997 induces apoptosis but not autophagy in prostate cancer cells. **a** DU145 and PC3 cells were treated with 60 nM CYT997 for the indicated times, and O_2_^·−^ release was determined by electrochemical biosensor. **b** DU145 and PC3 cells were treated with the indicated concentrations of CYT997 for 8 h, and ROS generation was determined by DCFH-DA staining. **c**, **d** DU145 and PC3 cells were treated with the indicated concentrations of CYT997 for 24 h, and cell lysates were collected for Western blot with the indicated antibodies (**c**), and apoptosis was determined by Cell Death Detection Elisa kit (**d**). *p < 0.05; **p < 0.01

Our pervious study has shown that sorafenib and other drugs approved for cancer treatment can cause mitochondrial dysfunction and increase intracellular oxidative stress [28]. We next examined whether CYT997 affects oxidative stress on prostate cancer cells. The results from electrochemical biosensors showed that more superoxide (O_2_^·−^) were released in DU145 cells in the presence of CYT997 (Fig. 2a). In contrast, there was no difference in release of O_2_^·−^ with or without CYT997 treatment (Fig. 2a). The cellular reactive oxygen species (ROS) evaluated by DCFH-DA further showed a significant increase of fluorescence in DU145 cells, but not in PC3 cells, following CYT997 exposure (Fig. 2b). These results are very consistent with the observations from electrochemical biosensors. To study whether CYT997 induces cell apoptosis, we determined the levels of cleaved PARP with or without CYT997 treatment. CYT997 led to a remarkable increase in cleaved PAPR in both DU145 and PC3 cells (Fig. 2c), which was confirmed with an increased apoptotic rate (Fig. 2d). Moreover, either DU145 or PC3 cells did not render autophagy in CYT997 treatment, as an evidence of no protein level changes in autophagic flux markers LC3B-II and p62 (Fig. 2c), suggesting that CYT997 induces apoptosis in prostate cancer cells via autophagy-independent mechanisms.

### Inhibition of Src augments the cytotoxicity of CYT997 in prostate cancer cells

**Fig. 3:**
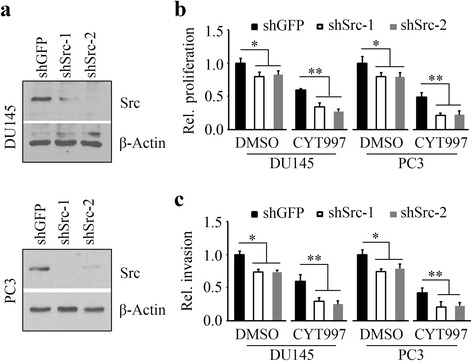
Knockdown of Src sensitizes prostate cancer cells to CYT997. **a** The shRNAs against Src (shSrc-1, shSrc-2) were used to deplete Src expression in DU145 and PC3 cells. The shRNA against GFP (shGFP) was used as a negative control. After transfection and infection, the puromycin-resistant stable cells were verified for knockdown by Western blot. **b**, **c** Src knockdown and control cells were treated with 60 nM CYT997. Cell proliferation was determined at 48 h after treatment by MTS assays (**b**), and cell invasion was determined at 24 h after treatment by Matrigel-coated modified Boyden chambers (**c**). *p < 0.05; **p < 0.01

As shown in Fig. 1d and Additional file 2: Figure S2, phosphorylation levels of Src were increased in CYT997-treated DU145 and PC3 cells compared with non-treated control cells. These data prompted us to investigate whether inhibition of Src expression improves the CYT997 efficacy. We depleted Src in DU145 and PC3 cells using a shRNA strategy (Fig. 3a) and determined the consequences of Src loss in the presence of CYT997. Knockdown of Src not only inhibited cell proliferation and invasion but also enhanced CYT997-induced suppression on these phenotypes (Fig. 3b, c), suggesting that blockage of Src may provide a clinical benefit for cancer patients following CYT997 treatment.

**Fig. 4:**
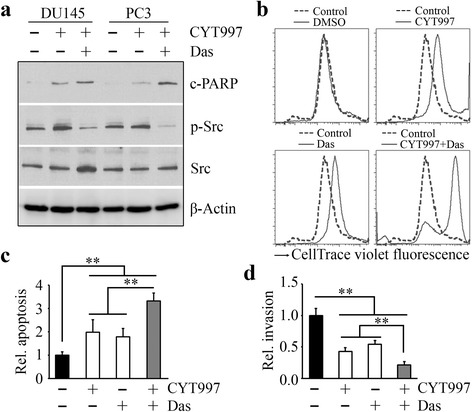
Dasatinib enhances in vitro cytotoxicity of CYT997 in prostate cancer cells. **a** DU145 and PC3 cells were treated with 60 nM CYT997 for 24 h, in the presence or absence of 50 nM dasatinib (*Das*). Cell lysates were collected for Western blot with the indicated antibodies. **b**–**d** PC3 cells were treated with 60 nM CYT997 for 24 h, in the presence or absence of 50 nM dasatinib. Cell death was determined by flow cytometry with Zombie Aqua staining (**b**), apoptosis was determined by Cell Death Detection Elisa kit (**c**), and cell invasion was determined by Matrigel-coated modified Boyden chambers (**d**). **p < 0.01

Dasatinib, an orally bioavailable synthetic small molecule inhibitor, can bind to and inhibit the growth-promoting activities of Src-family protein tyrosine kinases [21]. We thus co-treated prostate cancer cells with CYT997 and dasatinib. As would be expected, dasatinib attenuated CYT997-mediated phospho-activation of Src, with enhanced induction of cleaved PARP (Fig. 4a). Moreover, the combination of CYT997 and dasatinib acted more efficiently on cell death and apoptosis than either of the drugs alone (Fig. 4b, c). Figure 4d demonstrates that dasatinib augments the inhibitory effect of CYT997 on cell invasion.

### Dasatinib promotes the inhibitory effect of CYT997 on growth of prostate tumor xenograft in vivo

**Fig. 5:**
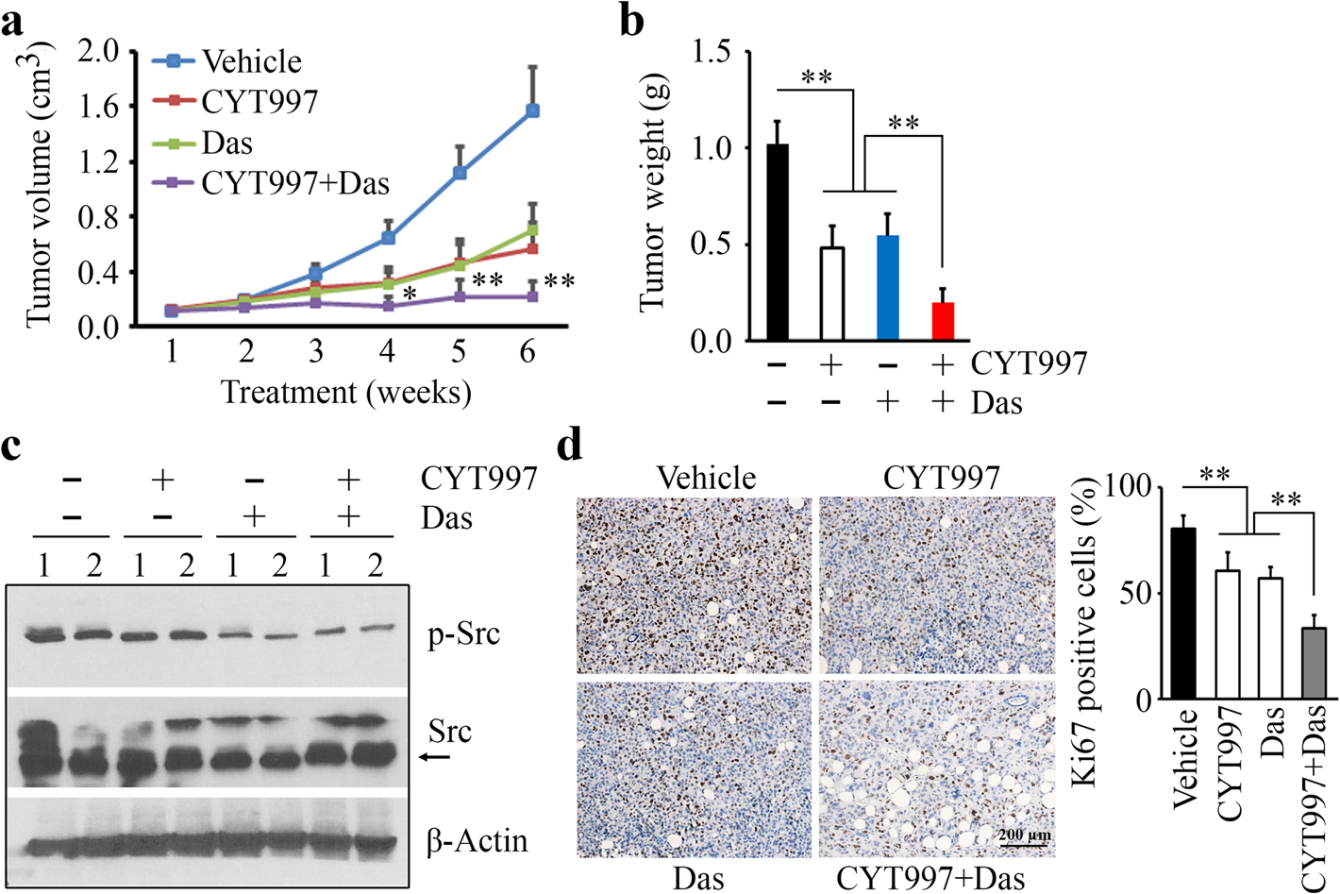
Combination of CYT997 and dasatinib exhibits greater inhibitory activity of prostate tumor growth than either treatment alone. **a**, **b** When the PC3-derived flank xenografts have been established, NSG mice were randomly divided into four groups (*n* = 5) for the indicated treatments. Tumor growth was measured by tumor volume (**a**), and tumor burden at the end of the experiment was calculated as tumor weight (**b**). **c**, **d** Mice were sacrificed on day 42 after treatment, and xenografts were dissected and removed for Western blot with the indicated antibodies (**c**) and IHC with Ki67 antibody (**d**). In **c**, *1* and *2* indicate the tumor samples from two different mice. In **d**, representative images of IHC are shown in the *left panel*, and quantitative data of staining intensity are shown in the *right panel* (*n* = 10). *p < 0.05; **p < 0.01

Based on these encouraging data, we used mouse tumor models to evaluate the synergistic effects of CYT997 and dasatinib on prostate cancer. The metastatic PC3 cells were implanted into the right flanks of NSG mice, and drug administration commenced on day 4 after implantation. Treatment with either CYT997 or dasatinib significantly reduced the size and weight of PC3-derived xenografts (Fig. 5a, b). The reduction in tumor growth was more significant in the mice receiving combined treatment compared with the mice receiving either drug alone (Fig. 5a, b). To determine whether Src signaling is involved in the synergistic drug effect, we examined Src activation using the cell lysates collected from the xenografts treated with CYT997 and dasatinib, alone or in combination. Western blot analysis showed that phosphorylation levels of Src were reduced in the dasatinib arm, but not in the CYT997 arm (Fig. 5c and Additional file 2: Figure S2). Dasatinib also blocked Src activation in CYT997 treatment (Fig. 5c and Additional file 2: Figure S2) and augmented CYT997-induced repression of tumor growth. IHC analysis with a Ki67 antibody confirmed that this co-treatment has a superior inhibitory effect on prostate tumor growth compared to monotherapy (Fig. 5d).

### Combination of dasatinib and CYT997 strongly suppresses prostate tumor metastasis in vivo

**Fig. 6:**
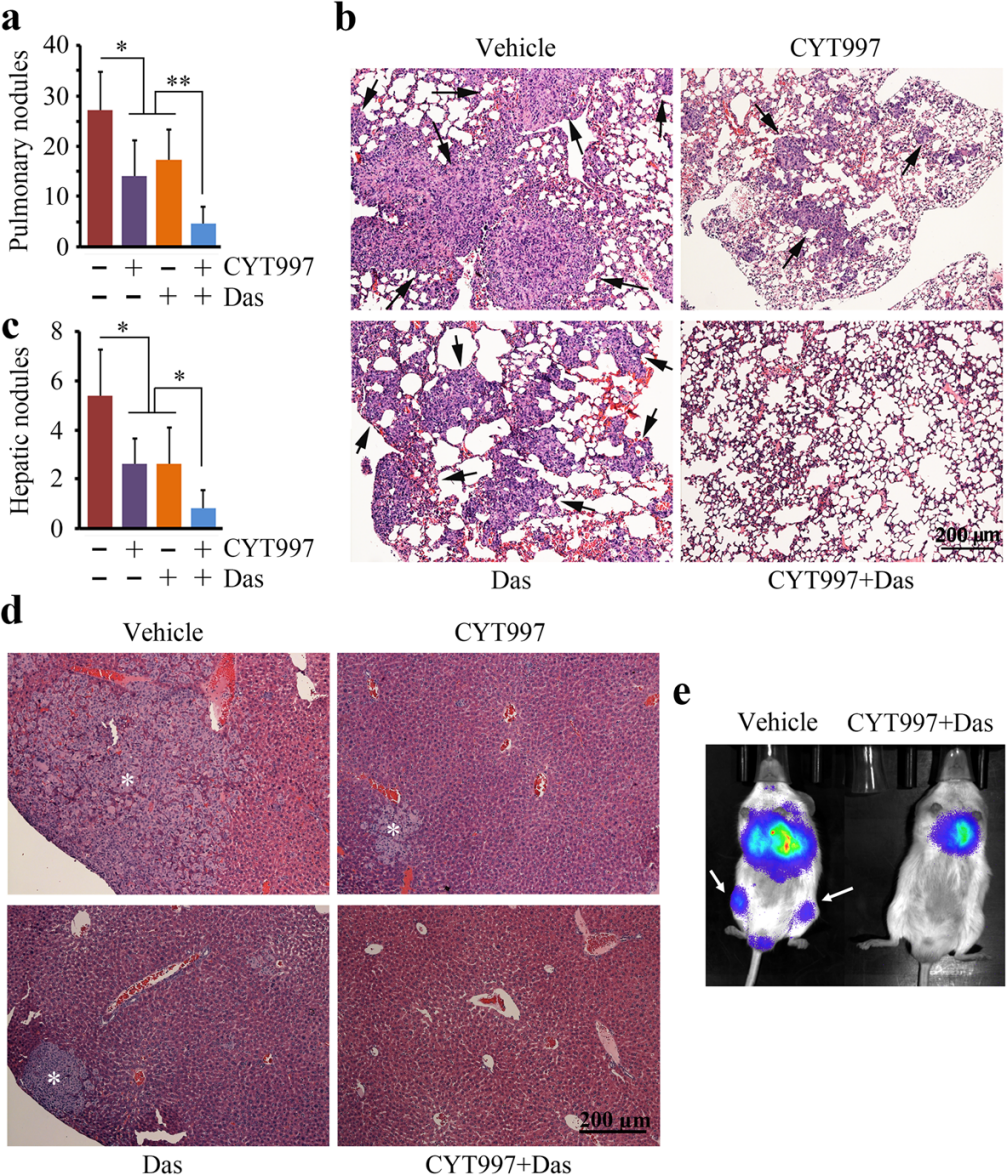
Dasatinib improves CYT997 efficacy in suppression of prostate cancer metastasis. **a**–**d** The tumor-bearing mice described in Fig. 5 were sacrificed on day 42 after treatment, and the lungs and livers were dissected and removed. The metastatic nodules on the pulmonary or hepatic surface were counted (**a**, **c**) (*n* = 5), and the tissue sections were stained with HE for pathological analysis (**b**, **d**). *Black arrows* in **b** indicate tumor foci in the mice lung, and *white stars* in **d** indicate tumor foci in the mice liver. **e** NSG mice were injected via intracardiac with 1 × 10^6^ luciferase-containing PC3 cells before treatment. Tumor progression was monitored by examining luminescence in Xenogen IVIS-200 In Vivo Imaging System. *p < 0.05; **p < 0.01

Our previous studies have demonstrated that tumor xenografts in NSG mice have potential to develop primary and metastatic tumors coincidently [29]. Therefore, we analyzed the mouse lungs and livers in xenograft model on day 42 after treatment. Incidence of metastatic nodules observed on the pulmonary and hepatic surface was significantly reduced in the mice treated with CYT997 or dasatinib compared with the mice treated with vehicle (Fig. 6a, c). The reduction in metastases in drug treatment was confirmed by histological analyses which showed decreased infiltrating tumors throughout the entire lung and liver (Fig. 6b, d). Combination of dasatinib and CYT997 displayed a stronger effect on suppression of metastasis than monotherapy, as evidenced by reduced nodule numbers on the pulmonary and hepatic surface (Fig. 6a, c) and relatively few, small tumor foci in the mice lung and liver (Fig. 6b, d). In order to detect the drug efficacy in bone metastasis, we generated an experimental metastasis mouse model by intracardiac injection of luciferase-containing PC3 cells. This model allowed for determination of the process of metastatic colonization at the bone and other organ sites followed by bioluminescence imaging. Notably, PC3 cells established colonies in the bone compartment of the hind leg (Fig. 6e). In contrast to these observations, synergistic treatment almost completely blocked dissemination of PC3 cells to other organs (Fig. 6e). These data suggest that dasatinib would exert an additive anticancer effect with CYT997 in treatment of patients with prostate cancer.

## Discussion

Microtubules are essential for cell growth, division, motility, intracellular trafficking, and the ability to adapt to a variety of shapes to interact with the environment [2]. Suppression of microtubule dynamics is a common mechanism of chemotherapeutic agents to block mitosis and kill tumor cells. CYT997, a novel anticancer MTA with a favorable combination of pharmacologic and pharmacokinetic properties and oral bioavailability, is currently undergoing clinical trials in a variety of cancer indications [9–11]. This work demonstrates the therapeutic efficacy of CYT997 in either cultured prostate cancer cells or mouse models of prostate cancer and reveals the possible mechanisms involved in drug action.

The therapeutic goal of cancer treatment has been to trigger tumor-selective cell death by apoptosis, and drug-mediated autophagy is increasingly recognized as an important factor in tumor apoptosis or survival [31]. Autophagic changes were not seen in the CYT997-treated prostate cancer cells, excluding the possibility that CYT997 promotes apoptosis in prostate cancer cells by accumulation of autophagy. Oxidative stress is one of the other contributing factors to apoptosis. CYT997 induces an increase of ROS levels and O_2_^·−^ release in DU145 cells, but not in PC3 cells. We also determined oxidative stress in AR-positive LNCaP cells with or without CYT997 treatment and observed the similar results seen in PC3 cells (data not shown). This discrepancy may be due to genetic background of the cell lines used in our study. One of our follow-up investigations is to discover possible molecular mechanisms mediating the apoptotic effect of CYT997.

SRC kinase activity has been found to be upregulated in a number of preclinical models of prostate cancer, and a potentially effective strategy to inhibit prostate cancer growth and metastasis is to target SRC kinases [21, 22]. Dasatinib inhibits multiple tyrosine kinases including SRC kinases, which are implemented to promote androgen independence in metastatic castration-resistant prostate cancer (mCRPC) that makes malignant cells unresponsive to therapies [32, 33]. Some of the common chemo drugs used to treat prostate cancer are MTAs. For example, docetaxel (Taxotere) stabilizes microtubules through binding to polymerized microtubules at the inner surface of the β subunit [34, 35]. As suggested by the phase III docetaxel plus dasatinib study, combination treatment is most effective in delaying and/or preventing skeletal metastases from prostate cancer [36]. However, no survival benefit was observed for the addition of dasatinib to docetaxel versus docetaxel/placebo [36, 37]. Despite the disappointing results seen from studies evaluating dasatinib in combination with other agents, clinical trials are needed to address whether dasatinib may be used as a single agent or in combination with chemotherapy mediated by novel MTAs.

There is a clear potential for CYT997 to be used in all prostate tumors carrying AR or not and perhaps all clinical stages of prostate cancer since it can greatly inhibit nearly all prostate cancer cell lines. Our data indicate that CYT997 possess highly potent cytotoxic activity through inhibiting the PI3K/AKT and MAPK oncogenic signaling cascades. However, CYT997 cannot block the Src pathway at the examined concentrations. Considering that monotherapy frequently fails to produce an adequate response and combining cancer drugs with disparate mechanisms of action is a feasible strategy to achieve high anticancer activities, we treated prostate cancer cells with CYT997 and dasatinib. This drug combination shows synergistically decreased cell proliferation and increased apoptosis, leading to distinctly improved anticancer activities in vitro and in vivo. In the present study, we generated two types of prostate cancer mouse models using PC3 cells. The combination of CYT997 and dasatinib inhibits both prostate tumor growth and metastasis in these mouse models, providing a rational basis for the clinical treatment for patients with prostate cancer. Since the majority of mCRPC contains AR or even overexpresses it, use of different cells such as C4-2B (a cell line can produce osteoblastic metastases in the lumbar spine) would provide another model to explore the mechanisms of drug action and add value to the obtained findings. Future trials should identify patient subpopulations with elevated activation of Src in prostate cancer and better define the biologic processes of prostate cancer progression that are specifically “Src-driven.” Combination treatment of CYT997 and dasatinib may be developed as a novel therapy to improve clinical outcomes for prostate cancer carrying high levels of activated Src.

## Conclusions

Taking the obtained findings together, this work shows for the first time that blockage of Src by dasatinib can augment the anticancer efficacy of CYT997 in prostate cancer development and progression. Our study explicates a rational basis of synergistic actions of CYT997 and dasatinib and provides preclinical data for possible clinical application of this novel therapeutic strategy. In future work, we will improve drug delivery system to target prostate cancer more effectively and precisely.

## Additional files

Additional file 1: Figure S1. CYT997 inhibits proliferation (a) and viability (b) of LNCaP derivative prostate cancer cell lines C4-2 and C4-2B. *p < 0.05; **p < 0.01. (DOCX 132 kb)

Additional file 2: Figure S2. Quantitative data of phosphorylation Src levels in in vitro (a, representative images are shown in Fig. 1d) or in vivo treatment (b, representative images are shown in Fig. 5c). *p < 0.05; **p < 0.01; *n* = 5. (DOCX 127 kb)

## Abbreviations

ABTS: 2,2′-Azino-bis(3-ethylbenzothiazoline-6-sulphonic acid)
AR: Androgen receptor
DCFH-DA: 2′,7′-Dichlorodihydrofluorescein diacetate
DMEM: Dulbecco’s modified Eagle’s medium
FBS: Fetal bovine serum
i.p.: Intraperitoneally
IHC: Immunohistochemistry
mCRPC: Metastatic castration-resistant prostate cancer
MTA: Microtubule-targeting agent
MTS: 3-(4,5-Dimethylthiazol-2-yl)-5-(3-carboxymethoxyphenyl)-2-(4-sulfophenyl)-2H-tetrazolium
NSG: NOD.Cg*-Prkdc* ^*scid*^ *Il2rg* ^*tm1Wjl*^ */SzJ*
O_2_^·−^: Superoxide
ROS: Reactive oxygen species
