# Supplementary material for: Augmentation of the anticancer activity of CYT997 in human prostate cancer by inhibiting Src activity
Source: J Hematol Oncol. 2017 Jun 12;10:118. doi: 10.1186/s13045-017-0485-0 (PMC5469135; doi:10.1186/s13045-017-0485-0)
Supplement: Supplementary file 1 — CYT997 inhibits proliferation (a) and viability (b) of LNCaP derivative prostate cancer cell lines C4-2 and C4-2B. *p < 0.05; **p < 0.01. (DOCX 132 kb) [file 13045_2017_485_MOESM1_ESM.docx]

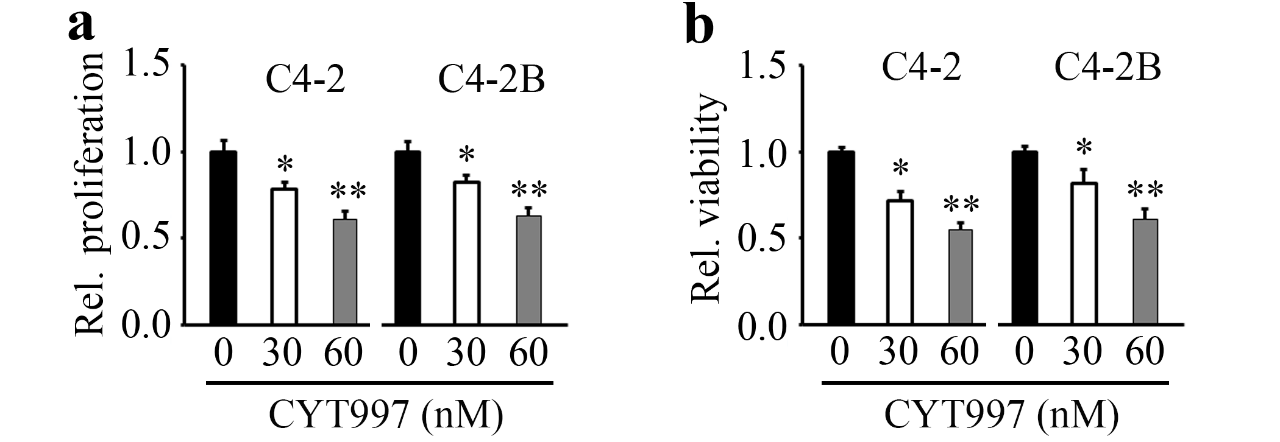


**Figure S1: CYT997 inhibits proliferation (a) and viability (b) of LNCaP derivative prostate cancer cell lines C4-2 and C4-2B.** **p*<0.05; ***p*<0.01.
