# Supplementary material for: Augmentation of the anticancer activity of CYT997 in human prostate cancer by inhibiting Src activity
Source: J Hematol Oncol. 2017 Jun 12;10:118. doi: 10.1186/s13045-017-0485-0 (PMC5469135; doi:10.1186/s13045-017-0485-0)
Supplement: Supplementary file 2 — Quantitative data of phosphorylation Src levels in in vitro (a, representative images are shown in Fig. 1d) or in vivo treatment (b, representative images are shown in Fig. 5c). *p < 0.05; **p < 0.01; n = 5. (DOCX 127 kb) [file 13045_2017_485_MOESM2_ESM.docx]

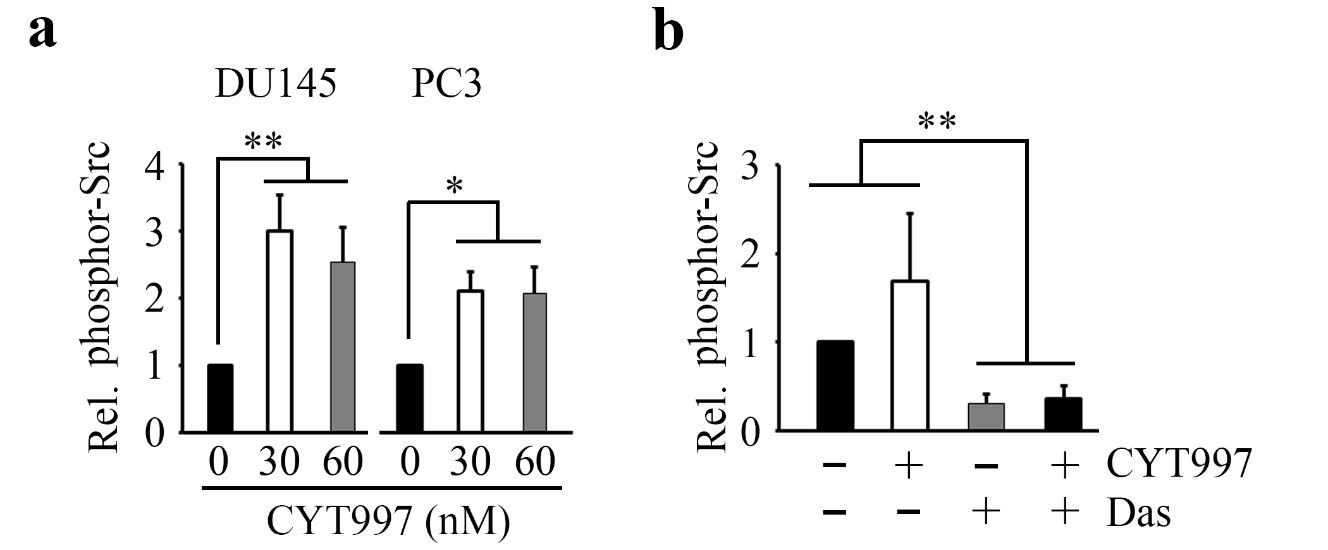


**Figure S2: Quantitative data of phosphorylation Src levels in *in vitro* (a, representative images are shown in Figure 1d) or *in vivo* treatment (b, representative images are shown in Figure 5c).** **p*<0.05; ***p*<0.01; n=5.
